# Abstract of Proceedings of the Buffalo Medical Association

**Published:** 1874-12

**Authors:** W. W. Miner

**Affiliations:** Secretary, pro tem.


BUFFALO

### Medical and Surgical Journal.

VOL XIV. DECEMBER, 1874. No. 5.

### Original Communications.

:o:

ART. I.—*Abstract of the Proceedings of the Buffalo Medical Association, Meeting held November 3d,* 1874. Reported by W. W. Miner, M. D., Secretary pro tem.

Dr. James P. White, President, occupied the chair.

Members present: Drs. Rochester, Johnson, Lothrop, Miner, Brush, Brecht, Hauenstein, Miner and Fowler.

In the absence of the Secretary, Dr. W. W. Miner was chosen secretary pro tem. The reading of the minutes of the last meeting were by vote dispensed with.

Dr. Lucien Howe was invited to be present and participate in the deliberations of the Society, until he should have oportunity to qualify for membership. It was voted that Dr. T. H. Boysen, be a member of the Society on his compliance with the usual regulations.

It was voted that an invitation be extended to the members of the Medical Class, at the College, who are preparing to graduate at the close of the present lecture course, to be present, if they desire, at the regular meetings of the Society.

It having been appointed at a previous meeting that a paper should be read, it was by invitation then presented by the writer, E. N. Brush, M. D., upon the Unity or Duality Theory in Syphilis.

Last June it was my good fortune to listen to a lengthy address by Prof. Gross, at Detroit, in which he took occasion to declare his adherence to the doctrine of the unity of the venereal virus. Since then, at meetings of this Association, I have heard remarks made which seem to indicate that some of its members were also inclined to the same opinion.

The term unity or duality of syphilis, is in itself a misnomer, for if there be but one characteristic veneral poison evincing itself in different ways, but still capable of producing constitutional effects, there is no such thing as duality; and if there be two distinct poisons, one always local, the other always constitutional, there can be but one *syphilitic poison*, and hence there is no duality of syphilis. Confining ourselves for the time being in the use of the word veneral to those two classes of disease manifested first by the appearance of the local sore, whether hard or soft chancre, we shall be in a position more definitely to discuss the question as to their unity or duality.

Something over twenty years ago, Bassereau, a pupil of Ricord's, advanced first the doctrine of duality. Bassereau, in his clinical observations, confined himself to the local or constitutional characteristics of the diseases referred to, and did not hamper his observations with any inquiries as to whether the chancre was hard or soft. By. a series of observations in which he practiced what is known as a system of confrontation, he proved, not that a woman having a chancre of a hard or soft base would communicate one of a similar character, but, that as the sore remained local or developed constitutional effects, it would remain local or *vice versa* in the persons contracting a sore from it, and they in turn would transmit a sore of a like character to others.

Bassereau and other well known authorities upon syphilis, assert that while local contagious ulcers upon the genitals, communicated by sexual intercourse, were well known to the ancients; the disease which we demoninate syphilis did not make its appearance until the latter part of the fifteenth century. It was at that time recognized as a new and distinct disease, and in the treatises of the times was described as such in separate and distinct chapters.

On the contary, Dr. Gross in his address, asserts his opinion, arrived at after long study, that syphilis has existed as a distinct disease from the earliest periods of society. He even goes so far back into the past as to cite Job as saying "My flesh is covered with putrid sores, (chapter VII.) and my bones are pierced with pains, in the night season my sinews take no rest" (chapter XXX). Certainly these are symptomatic of syphilis, but to say that they were produced by that disease is to say more than most men would like to on so slight history. As most English and French as well as many German treatises, state the fact positively that syphilis is of more modern origin than the contagious venereal ulcer of the ancient, we may, perhaps, consider that fact settled.

In his paper Prof. Gross alluded to the fact that syphilis was rapidly destroying the natives of the Sandwich Islands. At the time of the discovery of these Islands, syphilis did not exist on them, while a local ulcer, the result of impure sexual intercourse was common, resembling what is now called chancroid, or by many the non-infecting chancre. This seems to prove that these two varieties are different in character, and that one does not arise from the other. But historical proof at the best cannot be relied upon, and it is from clinical observations that we must draw our conclusions.

Fournier has defined chancroid as a specific malady, consisting in a peculiar ulcer which secretes a virulent auto-inoculable pus. It is a malady exclusively local, never giving rise to any symptom which can be referred to a constitutional infection. It will on the contrary be admitted by all that a man contracting a syphilitic chancre never escapes without constitutional effects.

The best diagnostic sign between chancroid and chancre, is the period of incubation, when it can be ascertained definitely. Chancroid appears in from twenty-four hours to eight days after impure connection. Chancre has a period of incubation varying from seventeen to thirty days; the average time in chancroid being about forty-eight hours, in chancre twenty-four days. The virus of syphilis once absorbed into the system, no amount of cauterization will prevent constitutional infection. Hill cauterized the abraded surface for a man who had had impure connection, and who tore his frænum, in the act, a slough separated, and the healthy surface cicatrized; in one month the scar indurated and syphilis followed. Diday cauterized a syphilitic chancre within six hours after its first appearance, and although a healthy surface was left and the sore healed promptly, general syphilis followed. There seemed to be no cases upon record in which the experiment has been tried to destroy the point of inoculation of syphilis upon healthy persons, but an analogy may be drawn from other cases. Clerc destroyed the point of vaccination in some children one hour after vaccination, but vaccinia followed, and a second attempt at vaccination failed to take. Aimé Martin destroyed the point of vaccination in seven children with Vienna paste at periods varying from one hour to twenty-four after the insertion of the virus; in only one out of the seven could vaccinia be again produced. The poison of glanders has been inoculated in horses, and in one minute the point cut out, but the disease followed.

The experience with chancroid has been widely different, except in a few obstinate cases it is found that cauterization produces the best results, a healthy sore resulting after the separation of the slough, which cannot be made to yield an auto-inoculable pus; moreover the application of certain fluids to the point of abrasion even after the lapse of several hours, will prevent the appearance of chancroid.

The most distinctive difference between the two is the fact that with but rare exceptions syphilis appears but once in a life time, while chancroid may be contracted as often as desirable. Lindmann inoculated himself 2,700 times, and other enthusiasts have been still more successful; and this brings up the subject of auto-inoculation, a diagnostic test of great value, it having been found that the pus of chancroids is capable of producing a similar sore upon the bearer, while it is with the greatest difficulty, and only after irritation of the sore, that a similar result can be obtained from chancre.

These two points have been the main argument in the doctrine of dualism, and although it has been found that a few cases have been observed where syphilis has resulted from a second exposure, the patient having once had the disease, the cases are so few that they do not overthrow the rule. It has also been found that in the few cases out of many hundred trials in which auto-inoculation has been successfully employed; from a chancre the resulting sore has been without a period of incubation, pustular in form and capable of being freely inoculated upon the bearer. This has been explained upon the theory of mixed chancre, the argument being that a person might have a sore which contained both the virus of chancre and chancroid.

From what has been passed in hasty review, it seems that the following points may be deduced:

1st. That two totally distinct diseases are represented by what are known as chancroid and chancre, one always local, the other constitutional; the virus of each being different, and each arising only from its kind.

2d. That the only unfailing diagnostic difference between chancroid and chancre is, that chancroid can be reproduced indefinitely upon the bearer by auto-inoculation.

3d. That chancroid appears in the course of about 48 hours; that chancre comes on after a period of incubation, the average of which is about 24 days.

4th. That a chancre cannot be auto-inoculated upon the bearer, nor can it be inoculated upon a person under the influence of syphilis, to this there are a few rare exceptions where auto-inoculation is successfully made from a chancre, it is only done from a mixed chancre, or from one which has been irritated.

5. The same rule holds true to the latter manifestation of sypilis, they are not auto-inoculable, but are readily transmissible to healthy persons.

The practical lessons to be derived from this are that it is not necessary to treat the person bearing a chancroid constitutionally, that only local treatment is desirable, the best being the employment of suitable caustics.

The majority of opinion seems also to lean to the theory that constitutional treatment is not beneficial before the secondary manifestations of syphilis. The syphilitic chancre generally is but a small affair and takes care of itself, very little local treatment being necessary.

Dr. Rochester—I would suggest further discussion on the doctrines presented in the paper, especially as to our adherence to them. My statement would be that the local manifestation presented by chancre may be serious in character, and it seems to me that great error in treatment might follow the teachings of the paper.

Dr. Miner—I have never seen anything in my experience that would cause doubt as to the duality of the virus of hard and soft chancres. Never saw constitutional effects from the chancroid. It is important to the profession, and to us personally, that the question of whether a chancroid can ever produce constitutional symptoms be authoritatively settled. Most of the authorities who write as well as those who observe, hold that chancroid is venereal, not syphilitic. Still we may not be able to determine the matter satisfactorily here in Buffalo, especially as there is diversity among the profession at large on this subject. For my part I have no more positive or deliberately formed opinion than that of the duality of the virus. I think the primary lesion of syphilis, local and unimportant as regards treatment. Mercury given for secondary symptoms helps the sore heal if it has not disappeared of itself previously. Do not think it becomes phagedenic; had not observed such result, and if ulcers did become serpiginous and extended, should think them of different nature from that of chancre.

Dr. Johnson—I consider simple chancroid a local affection, and my experience justifies this view. Have never known a syphilitic sore fail to produce constitutional symptoms. Believe in treating both these locally; cases coming to me have shown that the primary lesion of syphilis will progress to the formation of more extended or separate ulcerous patches. Cauterize a chancre early and it will prevent this ulcerative progress. If the point of infection is near the frenum especially, it should be cauterized as early as possible.

Dr. Rochester—I am confident that all feel obliged to Dr. Brush for the presentation of his scholarly and scientific article. While I was at Blackwells Island Hospital, Ricord advanced the duality theory, which I understand he has since retracted. Dr. Wm. Kelley, one of my associates in hospital duty at that time, had great opportunity for observation in venereal disease, and was a very reliable observer: shortly before his death, it was stated publicly by him that the duality theory was not good or safe to act upon. In some experiments made by us, inoculation of chancre on the thigh produced results very serious, almost fatal. The extensive ulceration thus caused was not erysipelatous or erythematous; and in this way I have seen some very troublesome sores many times caused. This would not be the case if the duality theory were correct. In a certain case that I had to deal with, a venereal sore which appeared eight days after impure connection, was immediately treated with caustics, and in another eight days was cured; it was accordingly considered non-infective. Two or three years afterwards secondary and tertiary syphilitic symptoms appeared, and ten years after syphilitic iritis. Dr. Abraham Dubois of New York, saw with me the case just mentioned of iritis, occurring ten years after improper intimacy, and in concurrence with me, he said he should insist that the copper-colored eruption, thinness of hair and other evidences, were of syphilitic character. I do not doubt that non-specific sores may occur, but relying upon my personal experience, it appears, no doubt, that serious local effects may primarily be produced by the syphilitic sore, and that afterwards, though at a remote period, the unmistakeable evidences of constitutional infection follow. These facts have been impressed upon me in a very authoritative manner.

Dr. Brush stated that Bumstead in a letter dated Paris, October, 1873, said that Ricord still maintained the distinction between hard and soft chancres, of chancre and chancroid, or the duality theory. Syphilitic infection sometimes follows where no noticeable abrasion has been manifest. As to the question whether it is advisable to give mercury before the secondary symptoms are pronounced, Van Buren & Keyes in their just issued work, remark that by such a course, a mercuro-syphilitic eruption sometimes follows which is less under control than the naturally occurring eruption, and cases of such character were brought to my attention a year or so since.

Dr. White—I would express thanks for the succinct and well presented paper. The history of syphilis, which Prof. Gross has presented comprehensively in his recent address before the American Medical Association, I do not consider of great practical moment, as quite variable views and differing modes of treatment have prevailed at different times and with different men. During the first fifteen of the forty years of my practice, I was so situated as to observe a great deal of venereal disease. From M. Ricord, whom I followed, a gentleman of American birth, and one who spoke English fluently, I learned that a non-constitutional sore if allowed to become indurated, would then become constitutional. While I do not care to enter upon the general consideration of treatment, I would state that my course in early practice was to cauterize early if possible, soft non-Hunterian chancres, indeed, I cauterized nearly all sores, both soft and hard. Of these a large portion never returned for later treatment, a small percentage did. In all the early part of my life I studied and tried to get along without mercury in the secondary and tertiary stages. I now give in the secondary period the proto-iodide of mercury in doses of from one-fourth to one grain, combined with conium; and in the tertiary, the deut-iodide of mercury in combination with iodide of potassium. In the hospitals of New York and of Europe, mercury has been found a most reliable agent. I formerly considered that constitutional infection by the primary lesion could be prevented. I think the treatment of these cases an interesting topic of discussion. Nursing may be the means of the conveyance of the disease: slight opportunities, such as are recounted by Prof. Gross, kissing, the old-time custom of transplanting teeth from one person's mouth to that of another, are means of its transmission as I have had occasion to witness.

Dr. Rochester gave the following history of a case whose occurrence was of considerable interest:

Was called to see a boy of five or six years who had been ill for two days; was having severe pain in his stomach and bowels, and was troubled with ineffectual attempts at vomiting. I presumed the trouble to have origin in his bowels, and prescribed castor oil and laudanum, which relieved the tenesmus he had been having, but he still complained of severe pain in his stomach and bowels, and would hold his bowels with his hands for relief. Still considering the trouble intestinal, I ordered opium and bismuth, with warm applications to the bowels. On the fifth or sixth day I noticed difficulty in the respiratory functions; there was considerable febrile movement, pulse 100. I detected a pleuritic friction sound at the posterior part of the right lung. Directed hot fomentations to chest, gave a diuretic, also iodide of potash. The patient progressed from bad to worse, and began to show signs of emaciation. On the third week I found the heart displaced to the left, its apex situated one inch to the left of the left nipple—no respiratory murmur was heard in the right lung, except in the clavicular regions with supplemental respiration in the other lung. Could the trouble be anything more than was revealed by these signs? Cough first appeared at the fourth week, then râles were heard close to the spine, and no normal resoance was to be obtained. The respiration was bronchial in character; the lungs were compressed to the utmost extent. Dr. Hill, of Otsego county, then visiting here, also Drs. Wetmore and Diehl, visited the patient with me, with a view to thoracentesis if thought proper. I noticed then a few hard nodules in the skin which might indicate scrofulous or tubercular enlargement; was not certain of empyema, though all agreed that fluid was present and that it must be pus. I took a hypodermic needle and pierced the seventh intercostal space behind, when blood came out; I then made puncture in the sixth; here nothing flowed. The case must be one of acute tubercular infiltration. Four or five days later the patient died, and post mortem examination, though at first denied, was afterwards hastily made; Drs. Diehl, Hopkins and Wetmore, with myself, being present. On lifting the sternum, the contents of the thorax bulged forwards; and the knife revealed the large cavity of an abscess filled with pus, occupying the superior and middle lobes of the right lung, while posteriorly the lung tissue was solidified. If the cavity had been evacuated, what would have been the effect? There was no evidence of tubercular infiltration surrounding the cavity; there was no hereditary tendency to phthisis in the family, and I do not regard this case of that character. The case of Mrs. Danforth, which was seen by me in connection with Dr. Miner some years since, was one which was somewhat similar in character to this; there being no cough present till a few days preceding death.

Scarlet fever was said to be prevalent in the city by Drs. Hauenstein and Lothrop.

Dr. ROCHESTER reported beside scarlet fever of somewhat severe character, several cases of diphtheria with albuminuria.

Dr. BOYSEN reported varicella.

Dr. T. M. JOHNSON was appointed to read a paper, the subject of the same to be announced in the usual notice of meeting sent members.

Adjourned.

